# Crystalline lens geometry from a clinical OCT-based biometer in pre-cataract surgery patients

**DOI:** 10.21203/rs.3.rs-9137111/v1

**Published:** 2026-04-12

**Authors:** Javier Rodriguez-Sanchez, Derick Owusu Ansah, Alberto de Castro, Gonzalo Velarde-Rodriguez, Yue Zhao, Ugur Celik, Mujdat Cetin, Dylan McBee, Jen-Li Dong, Yuli Lim, Li Wang, Douglas Donald Koch, Scott MacRae, Ramya Natarajan, Pravin Vaddavalli, Nicolás Alejandre-Alba, Eduardo Martinez-Enriquez, Susana Marcos

**Affiliations:** Spanish National Research Council; University of Rochester; Spanish National Research Council; Hospital Universitario Fundación Jiménez Díaz; University of Rochester; University of Rochester; University of Rochester; Baylor College of Medicine; Baylor College of Medicine; Baylor College of Medicine; Baylor College of Medicine; Baylor College of Medicine; University of Rochester; L V Prasad Eye Institute; L V Prasad Eye Institute; Hospital Universitario Fundación Jiménez Díaz; Spanish National Research Council; University of Rochester

**Keywords:** Optical Coherence Tomography, Crystalline Lens, Cataract Surgery, Aging, Lens Diameter

## Abstract

Understanding the interplay between crystalline geometry and refractive error is important to get insights into lens aging and guide the design and selection of artificial intraocular lenses that replace the aged crystalline lens in refractive lens exchange and cataract surgery. In this study we evaluated the relationships between the full geometry of the crystalline lens and age, axial length (AL), and refractive error in patients scheduled for cataract surgery. Optical Coherence Tomography (IOLMaster700, Zeiss) combined with custom-developed full crystalline lens shape reconstruction algorithms were applied to image 453 eyes from 327 subjects and quantify crystalline lens geometrical parameters, including thickness (LT), radius of the anterior and posterior surface (RAL, RPL), volume (LV), surface area (LSA), diameter (DIA) and equatorial plane position (EPP). Correlation and partial correlation analysis (controlling for age, AL, and refraction) were performed. Multiple linear regression models evaluated whether age and AL or refraction predicted LT, DIA, LV and LSA. Age was significantly correlated with LT, LV, LSA, DIA, EPP, and refraction, and these associations persisted after controlling for AL or manifest refraction spherical equivalent. AL was correlated with LT, EPP, DIA, RAL, and with DIA, RAL, RPL and LSA after adjusting for age. Overall, the lens expands axially and meridionally with age, increasing in thickness, diameter, volume and surface area, accompanied by a hyperopic shift. Longer eyes exhibited lenses with larger diameters and flatter anterior and posterior surfaces, partially offsetting myopia. These findings suggest that age- and growth-related lens remodeling contributes to refractive shifts.

## 1. Introduction

Understanding the variations in crystalline lens geometry with age, axial length (AL), and refractive error offers valuable insights into lens remodeling and age-related changes. When studied in a population with cataracts, this knowledge also provides useful information for the management of the disease. The geometric characteristics of the human crystalline lens have been previously studied in vivo [1–6] and ex vivo [7–11]. In vivo, measurements are typically obtained using optical imaging techniques such as Scheimpflug, Purkinje and optical coherence tomography (OCT) [3, 4], or via non-optical imaging techniques such as magnetic resonance imaging (MRI) [5, 6]. While MRI is the only technique to provide non-invasive images of the full shape of the lens, it is limited by its clinical availability and low resolution.

OCT enables rapid in vivo image acquisition with commercial instruments commonly employed in clinical practice. Swept-source OCT (SS-OCT) systems are able to quickly obtain 3-dimensional, high-resolution images of the crystalline lens, even in the presence of cataracts [12]. As with other imaging modalities, accurate estimation of anterior and posterior surface lens geometry requires correcting optical distortions introduced by refraction at the cornea and anterior lens surface [13]. Furthermore, direct visualization of lenticular structures is limited by the information visible through the pupil using these techniques. Previous studies have demonstrated and validated a method to accurately estimate and quantify the full shape of the crystalline lens in vivo from the lens area visible through the pupil [14–16].

In this study, we report quantitative geometrical characteristics of the full shape of the crystalline lens in-vivo as a function of age, axial length and refraction using a clinical OCT imaging system in a pre-cataract surgery patient dataset. The study provides valuable insights to understand the geometrical changes of the crystalline lens with age, axial length and refraction, as well as to improve intraocular lens selection and cataract surgery planning [17, 18].

## 2. Methods

### 2.1. Subjects

A patient registry, consisting of 453 eyes from 327 participants undergoing standard preoperative evaluation for cataract surgery, was compiled for this study. Participants were enrolled at the Flaum Eye Institute, University of Rochester, Rochester, NY, United States (n = 63 eyes, 35 participants), Baylor College of Medicine, Houston, TX, United States (n = 110 eyes, 103 participants), Fundación Jiménez Díaz University Hospital, Madrid, Spain (n = 200 eyes, 118 participants) and LV Prasad Eye Institute, Hyderabad, India (n = 80 eyes, 71 participants). Approval for this cross-sectional study was obtained from the Institutional Review Boards at the involved institutions. This study was conducted in accordance with HIPAA rules. Informed consents were obtained from all involved subjects after explanation of the nature and possible consequences of the study. This research adheres to the tenets of the Helsinki Declaration of 1964 and its later amendments.

Inclusion criteria comprised subjects older than 45 years who underwent OCT-biometry as part of the standard preoperative evaluation for cataract surgery (phacoemulsification with placement of an intraocular lens) at one of the four participating institutions. Exclusion criteria included history of previous ocular surgery, such as corneal refractive procedures (e.g., LASIK), pregnancy, and vulnerable-group designation.

### 2.2. Data collection

Age, sex, and manifest refraction were obtained from the participant’s medical record. Manifest refraction was converted to spherical equivalent (MRSE) for data analysis. For each participant, one or both eyes were imaged with IOLMaster700 SS-OCT (Carl Zeiss Meditec, Jena, Germany) in a darkened room. The instrument obtained six radial images and reported the pupil diameter and AL. Raw images were exported for custom 3-D analysis. The pixel size in the exported images was calculated using measurements of a grid of known dimensions at different axial positions. The calibration was validated through comparison with geometric measurements from custom-designed OCT systems [13, 19].

### 2.3. Crystalline lens full shape reconstruction

**Figure 1:**
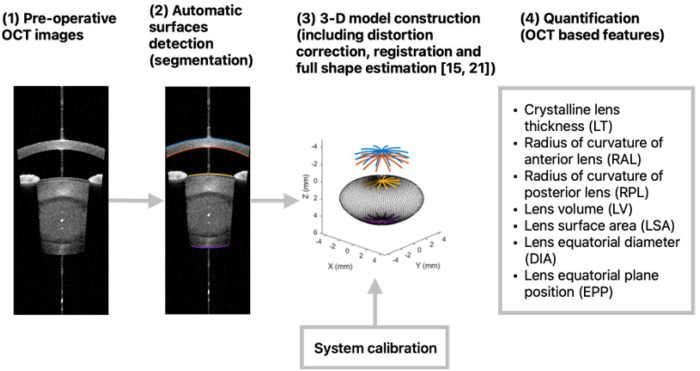
Crystalline lens full shape reconstruction from OCT images. OCT based crystalline lens features were quantified from the three-dimensional model, including the full shape of the crystalline lens.

Surface segmentation was performed using a dedicated deep learning algorithm, and a 3-D model of the crystalline lens was reconstructed with the data following fan and optical distortion correction, and registration, using previously described methodology (Fig. 1) [19, 20]. The lens thickness (LT) was obtained and the lens anterior (RAL) and posterior (RPL) radii of curvature were calculated through spherical fitting.

The full shape of the crystalline lens was estimated from the visible part through the pupil using the Eigenlenses method [15, 21], and the following parameters were obtained: lens volume (LV), lens surface area (LSA), lens equatorial diameter (DIA), and lens equatorial plane position (EPP), defined as the distance from the anterior lens apex to the equatorial plane. The LT/DIA ratio, which provides an estimate of the lens aspect ratio, was also calculated. To ensure accurate estimation of the crystalline lens full geometry, the main analysis included only eyes with pupil diameters exceeding 3 mm [22], with additional analyses performed on all eyes.

Measurements with incomplete data and those where automatic segmentation failed were excluded from the analysis. The number and percentage of eyes from male and female patients, and the mean and standard deviation (SD) age and refraction for the different study locations are shown in Table 1. A total of 333 eyes from 242 patients aged 45 to 92 years (mean 71 years, SD 9.22 years) were included in the primary analysis, corresponding to eyes with a pupil diameter greater than 3 mm. Comparable analyses were performed on two complementary datasets: (i) all pupil sizes, comprising 432 eyes from 316 patients (mean 71 years, SD 8.93 years), and (ii) one eye per patient with a pupil diameter ≥ 3 mm, 242 eyes (mean 70 years, SD 9.19 years). Subjects and ocular characteristics for each group are summarized in Table 2.

### 2.4. Statistical analysis

All analyses were performed using MATLAB (R2025b, Natick, Massachusetts, USA, The MathWorks, Inc.). Baseline characteristic analysis was performed for the different data locations to discard significant differences. The normality of each feature was assessed using the Shapiro-Wilk test. Since many of the features did not meet the assumption of normality, non-parametric analyses were performed. Specifically, Spearman’s correlation coefficient (r) was used to evaluate the relationship between OCT based crystalline lens features and age, AL and MRSE. Spearman’s partial correlation coefficient (ρ) was used to evaluate the relationship between crystalline lens parameters and age (controlling for AL and/or MRSE), AL (controlling for age), and MRSE (controlling for age). Multiple linear regression using the least squares method was used to determine how lens features were related to age and preoperative refraction, as well as to age and AL. Additional analyses were performed using the two complementary datasets: one that included eyes with pupil diameters smaller than 3 mm, and another limited to one eye per participant to account for the contribution of interocular relationships. For the latter, the eye with the larger measured pupil diameter was selected; when pupil sizes were equal, the right eye was chosen. A significance level of 5% was applied to all statistical tests.

## 3. Results

### 3.1. Age-related geometrical changes

**Figure 2:**
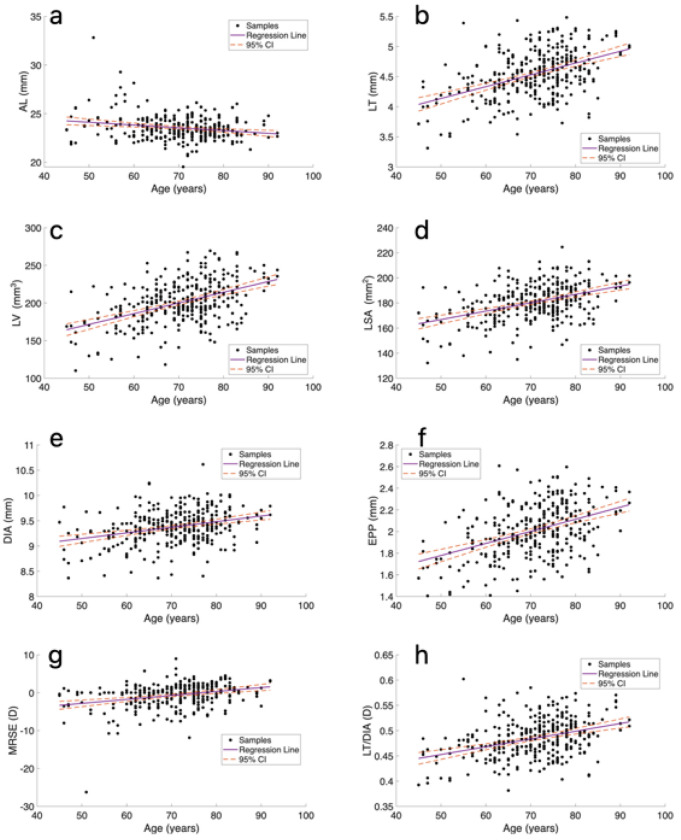
Lens parameters as a function of age and corresponding linear regression lines and 95% conifidence intervals (CI): a) AL (axial length), b) LT (lens thickness), c) LV (lens volume), d) LSA (lens surface area), e) DIA (lens diameter), f) EPP (equatorial plane position), g) MRSE (manifest refraction spherical error), and h) LT/DIA (lens thickness to diameter ratio).

The correlation coefficients of the lens parameters with age and the partial correlations when controlling for AL and refraction can be seen in Table 3, and significant correlations are presented in Fig. 2. The correlation of LT, LV, LSA, DIA, EPP and LT/DIA with age was significant, p < 0.05, (r = 0.42, 0.43, 0.39, 0.29, 0.39 and 0.34 respectively), and remained significant after controlling for AL (ρ = 0.41, 0.43, 0.41, 0.32, 0.38 and 0.32 respectively) and refraction (ρ = 0.41, 0.43, 0.39, 0.28, 0.39 and 0.33, respectively). This indicates that, on average, the lens became thicker and larger and increases its EPP with age. Eyes also tended to become more hyperopic (higher MRSE) with increased age (r = 0.29), and the correlation remained significant after controlling for AL (ρ = 0.25). Additionally, shorter AL was correlated with increasing age (r = −0.13); however, this correlation was no longer significant after controlling for refraction.

### 3.2. Axial length-related geometrical changes

**Figure 3:**
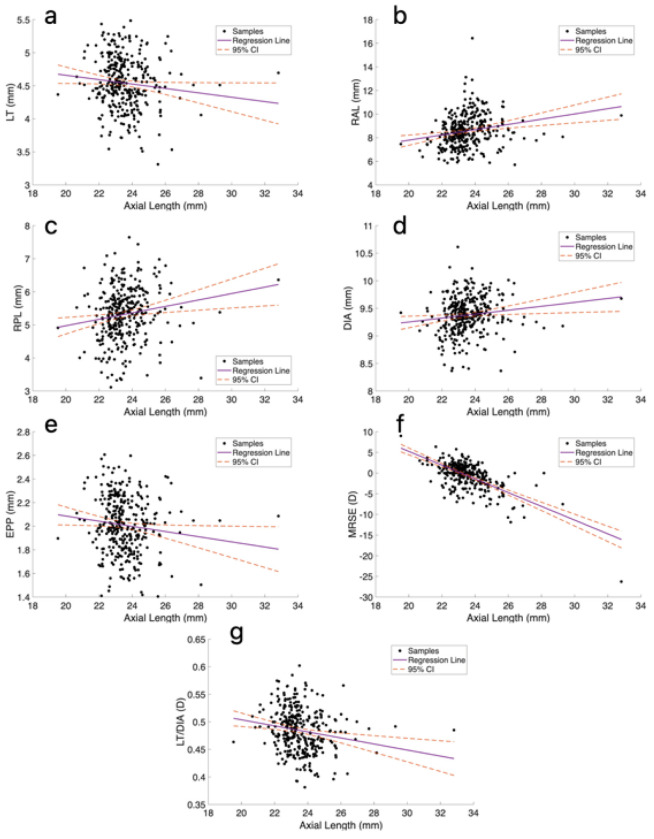
Lens parameters as a function of axial length and corresponding linear regression lines and 95% conifidence intervals (CI): a) LT (lens thickness), b) RAL (anterior radius of curvature), c) RPL (posterior radius of curvature), d) DIA (lens diameter), e) EPP (equatorial plane position), f) MRSE (manifest refraction spherical error), and g) LT/DIA (lens thickness to diameter ratio).

As detailed in Table 4 and illustrated in Fig. 3, RAL, RPL, and DIA were positively correlated with AL (r = 0.29, 0.20, and 0.19, respectively), and LT, EPP and LT/DIA were negatively correlated with AL (r = −0.13, −0.15 and −0.24, respectively). After controlling for age, the correlations of RAL, RPL, LSA, DIA and LT/DIA with AL were significant (ρ = 0.28, 0.21, 0.16, 0.24 and −0.20, respectively). Finally, AL showed strong negative correlation with MRSE (r = −0.51) even when controlling for age (ρ = −0.49).

### 3.3. Refraction-related changes

Table 5 and Fig. 3 show the results of the correlations with refractive error. AL was correlated with refractive error, with longer AL in myopic eyes (r= −0.51, p < 0.05) and this association remained statistically significant after controlling for age (ρ = −0.49). LT, LSA, LV, DIA, RAL, RPL, and EPP were not correlated with refraction. However, after controlling for age, both LV and LSA exhibited significant negative correlations with refraction (ρ = −0.13 and −0.14, respectively).

### 3.4. Multivariate analysis

Supplementary Table S7 shows the results of multiple linear regression analysis using least squares of the LT, DIA, LV and LSA of the crystalline lens with age and MRSE, as well as with age and AL. The variance explained (R^2^) by the multivariate model was, on average, 1.41 times higher than that explained by the univariate model using age (LT: 0.28 vs 0.21; DIA: 0.17 vs 0.09; VOL: 0.29 vs 0.22; LSA: 0.26 vs 0.18). The mean absolute error in the prediction was less than 6%, 3%, 9% and 5% (i.e., 0.27 mm, 0.24 mm, 19 mm^3^ and 9.89 mm^2^) for LT, DIA, LV and LSA respectively, when age and AL were used as predictors. A similar level of accuracy was achieved when age and refraction were used instead.

### 3.5. Other biometric relationships

LT showed a strong positive correlation with LV, DIA, and EPP (r = 0.84, 0.34 and 0.91, respectively, p < 0.05). Likewise, LV also tended to increase with increasing DIA (r = 0.78, p < 0.05). RAL was on one hand negatively correlated with LT and on the other, positively with DIA (r = −0.37 and 0.20, respectively, p < 0.05), whereas the RPL correlated positively with LV and DIA (r = 0.30 and 0.59, respectively, p < 0.05).

## 4. Discussion

This cross-sectional study made use of a previously established methodology by part of the authors for full crystalline lens shape estimation and biometric quantification using swept-source OCT data [15, 19–21]. The method has been applied to provide further insight into the quantitative differences observed in the crystalline lens geometry in a pre-cataract surgery patient group. To our knowledge, this is the first study that leverages the enhanced resolution and visualization capabilities of SS-OCT to quantify crystalline lens full shape parameters in relation to age, axial length and refraction in a cohort of pre-cataract surgery patients.

The baseline characteristics were similar across study locations as shown in Table 1. Further analyses were conducted applying the Kruskal-Wallis test to analyze if the geometrical features (DIA, LSA and VOL) measured in the different sites were similar in a subset of subjects with comparable age and refraction ranges. This analysis did not show significant differences between locations indicating that experimental data obtained in the four sites were consistent.

Muralidharan et al. used a custom-developed SS-OCT imaging technique to evaluate anterior segment biometric changes, including full crystalline lens parameters, as a function of refractive error and axial length in a cohort of young myopes (22–36 years of age) while controlling for age. Compared to our study in an older pre-cataract surgery group, they found higher changes in lens parameters with increasing axial length in a younger cohort: DIA (slope 0.15 vs 0.036), RAL (0.97 vs 0.22), RPL (0.36 vs 0.10), and LSA (2.82 vs 0.74) [23]. Furthermore, they reported an increase in AL, DIA, RPL and LSA with increased myopic refractive error, whereas in our older cohort, only AL, LSA and LV showed statistically significant correlations with refractive error when controlling for age. An additional study using SS-OCT in a pre-presbyopic population reported anterior lens curvature flattening with increased AL [24]. These findings suggest that the extent of the changes in lens geometry attenuate with advancing age. Since the correlations remain significant after controlling for axial length these age-related changes prevail over some of the lens remodeling associated to myopia development, which most likely occurred at an earlier age.

The observed increase in lens thickness (0.02 mm/year) with age is comparable to the results acquired using a laboratory-based SS-OCT in a younger population (0.015 mm/year) [25, 26], and also with results found with in-vivo MRI (0.02 mm/year) [5]. The MRI study also reported comparable trends in increasing lens diameter (0.007 vs 0.011 mm/year) with age, and a similar ratio of lens thickness to diameter (0.50 at age 65 vs 0.49 in our cohort) [5]. They hypothesized that the observed increase in lens diameter might have been confounded by tonic accommodation in their younger cohort. Our study on older, non-accommodating pre-cataract surgery subjects demonstrates similar age-related thickening of the lens, confirming that the crystalline lens enlarges both equatorially and axially with age. Moreover, we observed an increase in the lens thickness-to-diameter ratio with age, suggesting that the crystalline lens becomes progressively more spherical over time. An ex-vivo study in isolated lenses in the age range 20 to 56 years old also reported an age-related increase in LT (0.014 mm/year), DIA (0.023 mm/year), LV (1.67 mm^3^/year) and LSA (0.93 mm^2^/year), and a LT/DIA ratio of 0.45 [27]. The difference in the magnitude of the variations may be explained by the older age distribution of our cohort (45–92 years) compared with the younger subjects included in the ex-vivo study (20–56 years), as well as by the fact that isolated ex vivo lenses can be considered equivalent to fully accommodated lenses.

In our pre-cataract surgery population, eyes tended to become more hyperopic with age. Also, increased age was associated with a shorter AL in our main dataset; however, this correlation was no longer significant after controlling for refraction. These findings suggest that any apparent decrease in AL with age is primarily attributable to cross-sectional differences in refractive status. Similar apparent decreases in AL with age have been reported in previous studies. However, no physiological mechanism has been identified to explain such an age-related shortening of AL [28–30]. A literature analysis of AL variations found consistent age-related decreases in cross-sectional studies and suggested that this trend likely reflects a cohort effect. This effect may be related to global increases in education levels and body stature during the 20th century, and/or changes in lens refractive index that may artifactually influence AL measurements [29]. Consistent with this interpretation, the observed correlation between older age and shorter AL in our data may also reflect a cohort effect, whereby younger generations, exposed to greater environmental or near-work demands during ocular development, exhibit refractive changes leading to a longer and stable AL in adulthood.

We found that the radii of the anterior and posterior lens surfaces were not correlated with age. Assuming a homogeneous refractive index, these results would suggest that the refractive power of the crystalline lens may not change with age in our cohort. However, eyes were observed to become more hyperopic with age, despite a lack of known considerable changes in AL or corneal curvature with age [2]. This aligns with previous research on the lens paradox, a phenomenon in which the crystalline lens curvature and thickness are observed to increase with age, yet, paradoxically, the refractive power of the eye decreases and tends towards hyperopia [1–3]. It has been hypothesized that this observation is due to an age-related decrease in the equivalent refractive index of the crystalline lens counteracting the effect of crystalline lens steepening [31]. Changes in crystalline lens refractive index were not evaluated in this study; however, prior studies have reported decreases in the lens gradient of the refractive index (GRIN) power which may offset the crystalline lens geometrical changes and explain the paradox [32, 33]. Our study uses a homogeneous group refractive index to correct OCT images as this approximation has been shown to accurately reconstruct the posterior radius of curvature of the crystalline lens [34, 35]. Moreover, because statistically significant differences in lenticular biometry with refractive status were observed only for LSA and LV after controlling for age, we infer that the observed refractive changes with age are more likely driven by alterations in the internal lens structure or refractive index, rather than by geometric parameters alone.

In summary, our ^ndings suggest that with advancing age, the crystalline lens expands

axially and meridionally, adopting a more spherical shape. In eyes with longer axial length, the lens becomes thinner and flatter, increases in diameter, and assumes a more elliptical shape. The data also showed that refractive error was directly correlated with AL and with LSA and LV only after controlling for age.

This study presents a few limitations. One is the accuracy of the estimation of the full geometry of the crystalline lens in small pupil sizes. Subjects with a pupil diameter smaller than 3 mm were excluded from the main analysis, as the dispersion of estimated full crystalline lens shape parameters (e.g., diameter) increased significantly below this threshold, indicating reduced accuracy in model reconstruction [22]. Also, we have shown in previous work that the LV, DIA, and EPP estimation error did not change significantly for different pupil sizes above 4 mm [14]. A complementary analysis was performed using a dataset that also included eyes with a pupil diameter smaller than 3 mm (see Supplementary tables S1-S3-S5), and all results remained consistent except for the partial correlation of RAL with age when controlling for refraction, and for RPL with age when controlling for AL that were significant. This suggests that, despite potential estimation errors, the observed trends persisted without discarding eyes with small pupils.

Another limitation involves the inclusion of both eyes from the same subject, which could potentially introduce inter-eye correlations and bias the statistical analysis. To account for this effect, a complementary analysis was conducted using a subset of data that included only one eye per participant (see Supplementary tables S2-S4-S6). The results from this reduced dataset remained consistent with those obtained from the main cohort, except for the correlations between AL with age and between LT and EPP with AL that were not found to be significant. Despite these differences, the statistically significance of most correlations remained stable and the similarity in the slopes indicated that the inclusion of both eyes did not substantially affect the main findings of the study.

The study included subjects with different types of cataracts, clinically evaluated using the LOCS III scale. The distribution of cataract type and grade was similar across centers: 96% of the 432 lenses presented nuclear cataracts (mean NS = 3.0), 42% had cortical cataracts (mean C = 1.7), and 21% presented subcapsular cataracts (mean PSC = 2.3; mean ASC = 1.0). Although some cataract types are known to induce changes in refractive error [36], these changes are typically small (generally below 0.5 D), and therefore are not expected to represent a limitation for the present study.

This study’s cross-sectional analysis may limit the interpretation of temporal changes in the crystalline lens geometry. Future studies will focus on tracking changes in the crystalline longitudinally, to provide further insights into the natural progression of these age-related changes.

Furthermore, this novel modality for quantitatively assessing crystalline lens geometry may be useful in other contexts, such as assessing lens changes in accommodation, myopia development, customizing presbyopia correction alternatives, determining effective lens position after cataract surgery, and predicting intraocular lens tilt.

## Supplementary Material

This is a list of supplementary files associated with this preprint. Click to download.

• Supplementarymaterial.docx

## Figures and Tables

**Table 1:** Baseline characteristics across study locations

	ROC	BCM	FJD	LVPEI
**Sex**				
**Male (%)**	35 (53.8%)	36 (34.6%)	80 (41.7%)	39 (54.9%)
**Female (%)**	30 (46.2%)	68 (65.4%)	112 (58.3%)	32 (45.1%)
**Age**				
	69 ± 8 years	71 ± 8 years	74 ± 9 years	64 ± 8 years
**Refraction**				
	−1.06 ± 3.19 D	−0.84 ± 4.20 D	−0.28 ± 3.06 D	−1.29 ± 2.94 D

ROC: Flaum Eye Institute, University of Rochester, Rochester, NY, United States (n = 65 eyes, 35 participants); BCM: Baylor College of Medicine, Houston, TX, United States (n = 104 eyes, 100 participants); FJD: Fundación Jiménez Díaz University Hospital, Madrid, Spain (n = 192 eyes, 118 participants); LVPEI: LV Prasad Eye Institute, Hyderabad, India (n = 71 eyes, 63 participants).

**Table 2:** Baseline characteristics per group of analysis

	All pupils (n = 432)	Pupils ≥ 3mm (n = 333)	One eye per patient & Pupils ≥ 3mm (n = 242)
**Sex**			
**Male (%)**	190 (43.78%)	141 (42.3%)	104 (43%)
**Female (%)**	244 (56.22%)	192 (57.7%)	138 (57%)
**Age**			
	71 ± 9 years	71 ± 9 years	70 ± 9 years
**Refraction**			
	−0.70 ± 3.38 D	−0.66 ± 3.3 D	−0.75 ± 3.48 D

**Table 3:** Lens parameters correlations with age

	Correlation with age			Partial correlation with age controlling for AL		Partial correlation with age controlling for refraction	
	Pupils ≥ 3 mm (n = 333)						
Feature	Slope	r	p-value	ρ	P-value	ρ	p-value
**AL**	−0.028	−0.13	0.01*	-	-	−0.02	0.77
**LT**	0.02	0.42	1.3e-15*	0.41	7.71e-15*	0.41	1.01e-13*
**RAL**	−0.015	−0.07	0.20	−0.03	0.55	−0.09	0.12
**RPL**	0.003	0.03	0.59	0.06	0.29	0.02	0.78
**LV**	1.423	0.43	4.07e-16*	0.43	1.16e-16*	0.43	8.44e-15*
**LSA**	0.668	0.39	8.48e-14*	0.41	5.51e-15*	0.39	1.11e-12*
**DIA**	0.011	0.29	1.06e-07*	0.32	2.19e-09*	0.28	6.11e-07*
**EPP**	0.011	0.39	1.82e-13*	0.38	1.23e-12*	0.39	2.1e-12*
**MRSE**	0.104	0.29	2.37e-07*	0.25	1.49e-05*	-	-
**LT/DIA**	0.0015	0.34	1.29e-10*	0.32	1.63e-09*	0.33	2.64e-09*

Correlation of AL (axial length), LT (lens thickness), RAL (radius of curvature of the anterior lens surface), RPL (radius of curvature of the posterior lens surface), LV (lens volume), LSA (lens surface area), DIA (lens diameter), EPP (equatorial plane position), MRSE (refraction spherical equivalent) and LT/DIA (lens thickness to diameter ratio) with age. Slope is given in mm/year for AL, LT, RAL, RPL, DIA and EPP, mm^2^/year for LSA, mm^3^/year for LV, D/year for MRSE and year^−1^ for LT/DIA. Asterisks designate statistically significant correlations.

**Table 4:** Lens parameters correlations with axial length

	Correlation with AL			Partial correlation with AL controlling for age	
	Pupils ≥ 3 mm (n = 333)				
Feature	Slope	r	P-value	ρ	P-value
**LT**	−0.033	−0.13	0.02*	−0.08	0.15
**RAL**	0.222	0.29	7.2e-08*	0.28	1.54e-07*
**RPL**	0.097	0.20	0.0002*	0.21	0.0002*
**LV**	0.418	0.03	0.61	0.09	0.09
**LSA**	0.740	0.09	0.20	0.16	0.004*
**DIA**	0.036	0.19	0.0004*	0.24	7.84e-06*
**EPP**	−0.022	−0.15	0.008*	−0.10	0.06
MRSE	−1.663	−0.51	2.07e-21*	−0.49	1.26e-19*
**LT/DIA**	−0.006	−0.24	1.5e-05*	−0.20	0.0002*

Correlation of LT (lens thickness), RAL (radius of curvature of the anterior lens surface), RPL (radius of curvature of the posterior lens surface), LV (lens volume), LSA (lens surface area), DIA (lens diameter), EPP (equatorial plane position), MRSE (refraction spherical equivalent) and LT/DIA (lens thickness to diameter ratio) with AL (axial length). Slope is given in mm/mm for LT, RAL, RPL, DIA and EPP, mm for LSA, mm^2^ for LV, D/mm for MRSE and mm^−1^ for LT/DIA. Asterisks designate statistically significant correlations.

**Table 5:** Lens parameters correlations with refraction

	Correlation with refraction			Partial correlation with refraction controlling for age	
	Pupils ≥ 3 mm (n = 333)				
Feature	Slope	r	P-value	ρ	P-value
**AL**	−0.2576	−0.51	2.07e-21*	−0.49	1.26e-19*
**LT**	0.0015	0.05	0.34	−0.07	0.2
**RAL**	−0.0139	-−.05	0.34	−0.03	0.6
**RPL**	−0.0120	−0.01	0.90	−0.01	0.82
**LV**	−0.1704	0.01	0.93	−0.13	0.02*
**LSA**	−0.1167	−0.01	0.9	−0.14	0.02*
**DIA**	−0.0035	−0.01	0.82	−0.10	0.07
**EPP**	−0.0001	0.05	0.36	−0.07	0.22
**LT/DIA**	0.0004	0.08	0.18	−0.02	0.68

Correlation of AL (axial length), LT (lens thickness), RAL (radius of curvature of the anterior lens surface), RPL (radius of curvature of the posterior lens surface), LV (lens volume), LSA (lens surface area), DIA (lens diameter), EPP (equatorial plane position) and LT/DIA (lens thickness to diameter ratio) with refraction. Slope is given in mm/D for AL, LT, RAL, RPL, DIA and EPP, mm^2^/D for LSA, mm^3^/D for LV, and D^−1^ for LT/DIA. Asterisks designate statistically significant correlations.

## Data Availability

The datasets analyzed during the current study are available in a GitHub repository: https://github.com/JavierRodriguez-IO/Crystalline-lens-geometry-from-a-clinical-OCT-based-biometer-in-pre-cataract-surgery-patients
